# Potential of Fungal Endophytes Isolated from Pasture Species in Spanish Dehesas to Produce Enzymes under Salt Conditions

**DOI:** 10.3390/microorganisms11040908

**Published:** 2023-03-31

**Authors:** Carlos García-Latorre, Sara Rodrigo, Oscar Santamaría

**Affiliations:** 1 School of Agricultural Engineering, University of Extremadura, Avda. Adolfo Suárez s/n, 06007 Badajoz, Spain; cgarcialn@unex.es; 2 Indehesa Research Institute, Campus de Badajoz, University of Extremadura, Avda. de Elvas s/n, 06006 Badajoz, Spain; saramoro@unex.es; 3 Department of Plant Production and Forest Resources, University Institute for Research in Sustainable Forest Management (iuFOR), University of Valladolid, Avda. Madrid 57, 34004 Palencia, Spain

**Keywords:** secondary metabolites, by-products, endophytic fungi, agrifood industry, halotolerance

## Abstract

Endophytic fungi have been found to produce a wide range of extracellular enzymes, which are increasingly in demand for their industrial applications. Different by-products from the agrifood industry could be used as fungal growth substrates for the massive production of these enzymes, specifically as a way to revalorize them. However, such by-products often present unfavorable conditions for the microorganism’s growth, such as high salt concentrations. Therefore, the objective of the present study was to evaluate the potential of eleven endophytic fungi—which were isolated from plants growing in a harsh environment, specifically, from the Spanish dehesas—for the purposes of the in vitro production of six enzymes (i.e., amylase, lipase, protease, cellulase, pectinase and laccase) under both standard and salt-amended conditions. Under standard conditions, the studied endophytes produced between two and four of the six enzymes evaluated. In most of the producer fungal species, this enzymatic activity was relatively maintained when NaCl was added to the medium. Among the isolates evaluated, *Sarocladium terricola* (E025), *Acremonium implicatum* (E178), *Microdiplodia hawaiiensis* (E198), and an unidentified species (E586) were the most suitable candidates for the massive production of enzymes by using growth substrates with saline properties (such as those found in the many by-products from the agrifood industry). This study should be considered an initial approach by which to further study the identification of these compounds as well as to develop the optimization of their production by directly using those residues.

## 1. Introduction

The term endophyte includes microorganisms, mainly fungi and bacteria, which colonize the internal tissues of plants without causing any visible disease symptoms [1]. Although many of these endophytes have been described to have a symbiotic role [2], many others are opportunistic species that are waiting for plant senescence in order to take advantage of the plant tissues’ colonization [3]. In the case of endophytic fungi, this colonization is carried out through extracellular enzymes that, together with the production of many other metabolites, are capable of degrading the cell wall of the plant [4] and counteracting its chemical defenses [5,6]. Different groups of these enzymes, such as pectinases, xylanases, cellulases, lipases, proteases, or phenol oxidases have been described to be involved in this process [7]. These compounds have been found to be also involved in the plant immune system by eliciting defense mechanisms against pathogens [8], as well as in the growth status of the host, whereby nutrient uptake through the roots is enhanced [9].

Besides their ecological roles, all these enzymes are valuable supplies that are used in numerous food, pharmaceutical, or paper industries [10,11,12]. For example, pectinases play an important role as fining agents in the juice and wine industries by enhancing the clarification process and by improving the overall quality of the final product [11]. Similarly, fungal amylases have increasingly been used for starch hydrolysis in the production of simple sugars and syrups, as well as in the optimization of the beer mashing process [5,13]. Microbial proteases and lipases are also used in the food industry, specifically for the stabilization of dried products in the milk industry [11] and for extending the shelf life of bakery products [14]. These enzymes have other important applications. For example, in the cosmetic industry—as in the case of proteases—they are used as a component in creams for the removal of dead cells [15]; lipases, for obtaining fatty amides [16]; or laccases, which are used in the preparation of hair dyes [17]. In the case of the paper industry, fungal laccases are used for the polymerization of fibers [11] and cellulases in order to harden the final product [18], which may allow a huge amount of energy to be saved during the paper-making process [19].

Although these enzymes can be obtained from many sources, those which are produced by fungi and bacteria have been described to be more stable during their processing [20]. However, only around twenty of these microbial enzymes, such as those obtained from different *Aspergillus* species, are produced on an industrial scale [21]. Therefore, the use of endophytes, in this case fungal endophytes, might provide a suitable alternative by which to try to expand the range of organisms to massively produce these kinds of enzymes. This massive production would need the utilization of a vast amount of substrate to grow the enzyme-producing fungi. In this regard, the use of by-products from the agrifood industry may allow one to obtain the amount of substrate required while, at the same time, offloading those initially valueless wastes [22]. However, these residues are usually not particularly favorable for fungal growth, as they may contain tannins, phenols, and other substances with biocide activity [23]. In addition, their chemical properties, such as salinity, might not be the most appropriate for their development [24]. However, this negative consideration regarding the unfavorable properties of such substrates might have a positive counterpart: the endophytic fungi that are capable of growing under these difficult conditions may produce a wider variety and greater number of these enzymes, as the link between enzyme production and unfavorable growth conditions has been already demonstrated [25]. Several agro-wastes and by-products—with limiting characteristics for fungal growth, such as salinity—have already been tested for use as substrates for fungal inoculation, as well as in the production of bioactive compounds, as is the case for olive-mill wastewaters [26]. However, the scientific literature in this matter is still quite limited.

Due to the role of such substances in the health status, performance, and growth of the plant host, the fungal endophytic species that are isolated from plants growing under harsh conditions, such as drought and salinity, may be more suitable to produce to a greater extent these kinds of compounds [27]. Spanish dehesas are grasslands with scattered trees and a well-developed herbaceous understory, which are used mainly for the extensive rearing of livestock. Furthermore, they are characterized by low and irregular rainfall as well as by soil with low fertility and, eventually, salinity [28,29]; these conditions are particularly unfavorable for the plant’s growth. For this reason, fungal endophytes which are isolated from the pastures growing in these ecosystems could be especially suitable candidates in terms of producing such substances. In order to identify the endophytic strains with the potential to produce enzymes, especially when grown in saline media, the use of qualitative tests could be considered one of the best options, as they might allow one to examine a wide range of isolates for several types of enzymes [30].

Thus, the aim of this study was to evaluate the potential of several fungal endophytes that were isolated from the pastures of dehesas in order to produce enzymes with applications in the fields of agrifood and other industries. A second goal was to evaluate their capacity to colonize and produce enzymes in a saline environment, such as those that are provided by many of the industrial by-products being considered. This would be a first step to assess if they could be further used as solid-state fermentation substrates that would be utilized to massively produce these enzymes.

## 2. Materials and Methods

### 2.1. Fungal Material

Eleven fungal strains, previously isolated from different healthy plants collected from the dehesas of Extremadura (in the southwest of Spain), were selected for the study (Table 1). The identification of these eleven fungi was first attempted morphologically by means of their reproductive structures, and then at a molecular level through the comparison of their ITS region sequence with those included in two databases, GenBank (www.NCBI.nlm.nih.gov, accessed on 13 December 2022) and UNITE (https://unite.ut.ee, accessed on 13 December 2022), while using a BLAST search [31]. A more exhaustive explanation regarding the identification and the species assignation process can be consulted in the works of Lledó et al. [32] and Santamaría et al. [33]. The endophytic strains were selected according to their frequency of isolation from the original plant hosts or through the observation of bioactivity in preliminary assays [34,35].

### 2.2. Evaluation of Extracellular Enzymatic Activity

**Figure 1:**
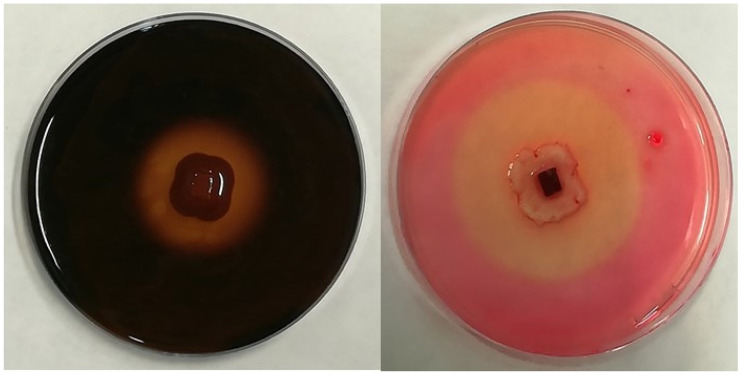
Examples of the hydrolysis halo observed in the present study for positive results of amylolytic activity (**left**) and cellulolytic activity (**right**).

The six most used enzymes by industry (i.e., amylase, cellulase, laccase, lipase, pectinase, and protease) were chosen in order to qualitatively evaluate the extracellular enzymatic activity of the selected fungi [36]. For that purpose, a 5 mm diameter plug of mycelia (obtained from an actively growing 7-day-old colony on potato dextrose agar medium; PDA) was placed in the center of a Petri dish containing the specific culture medium necessary to assess the production of each enzyme, as indicated below. Agar plugs without mycelia were placed in Petri dishes with the specific media to be used as a negative control. Once inoculated, the plates, prepared in triplicate, were later incubated for 7 days at 23 °C, as this is considered the optimal growth temperature for the selected fungi. After the incubation period, the formation of a hydrolysis halo around the colony was considered an indicator of enzymatic activity (Figure 1). In positive cases, the extension of both the colony and the clear area around it were measured to calculate the solubilization index (SI) as SI = (colony diameter + halo zone diameter)/colony diameter [37]. The specific media for the identification of each enzyme activity is described as follows.

Amylase activity was assessed by using a yeast malt agar medium (malt extract 10.0 g; yeast extract 6.0 g; D-glucose 4.0 g; agar 20 g; in 1 L of distilled water; pH 6.3), which was amended with a 1% soluble starch (Panreac Química SLU, Castellar del Vallès, Barcelona, Spain). After the incubation time, plates were flooded with a 1% iodine solution, which allowed us to identify the clear halo surrounding the colony in the case of positive activity [38].

In the case of cellulase activity, Petri dishes with a yeast malt agar medium were supplemented with 0.5% Na-carboxy-methylcellulose (Sigma-Aldrich, San Luis, MO, USA). After the growing period, plates were first flooded with 0.2% Congo Red (Merck KGaA, Darmstadt, Germany) and then with a 1 M NaCl solution, which allowed us to identify positive cellulase activity through the hydrolysis halo [35,38,39].

Laccase activity was detected by using glucose yeast extract peptone agar medium (glucose 5.0 g; peptone 5 g; yeast extract 3.0 g; agar 20.0 g; in 1 L of distilled water; pH 6.8) with 0.05g 1-napthol L-1 (pH 6.0) (Sigma-Aldrich, San Luis, MO, USA). As the fungus produced the enzyme, the colorless medium turned blue due to oxidation of the substrate [20].

For lipase activity, the endophytes were grown in a peptone agar medium (peptone 10.0 g; NaCl 5.0 g; CaCl_2_·2H_2_O 0.1 g; agar 16.0 g; in 1 L of distilled water; pH 6.0) supplemented with 1% Tween 20 (Merck KGaA, Darmstadt, Germany) which was sterilized separately and added before pouring onto the plates. The hydrolysis halo was directly visible as the fungi grew if they exhibited lipase activity [36].

Pectinolytic activity was determined by growing the fungi in a pectin agar medium (pectin 5 g; yeast extract 1 g; agar 20 g; in 1 L of distilled water; pH 5.0). After the incubation period, the plates were flooded with a 1% aqueous solution of hexadecyl trimethylammonium bromide (Panreac Química SLU, Castellar del Vallès, Barcelona, Spain) in order to detect the clear zone that formed around the fungal colony in the case of positive activity [20].

Finally, protease activity was evaluated by using a casein hydrolysis medium (skimmed milk powder 28.0 g; peptone 5.0 g; yeast extract 2.5 g; glucose 1.0 g; agar 20 g; in 1 L of distilled water; pH 7.0). A clear zone, directly visible around the colony, confirmed positive activity [39].

### 2.3. Halotolerance Test

To assess the capacity of the fungi to grow under salt stress conditions, Petri dishes were prepared with a PDA medium adjusted to different concentrations of NaCl (100, 200, and 500 mM) [40]. Likewise, plates containing the same medium but without NaCl (0 mM) were introduced as the control. After placing an actively growing plug (ø = 5 mm) of mycelia from each of the endophytes on the center of the plate, its radial growth was measured 9 days later in order to assess the maximum saline concentration that they were able to tolerate. All samples were analyzed in triplicate, and the results were expressed as the cm of radial growth.

### 2.4. Evaluation of Enzymatic Activity under Salt Stress Conditions

Once both the qualitative enzymatic activity and the halotolerance of fungal strains were assessed, another test was conducted in order to evaluate their potential to produce the different enzymes under salt stress. The selected endophytes were placed again in the basic agar media with the specific substrate for the corresponding enzyme, but in this case, they were adjusted to a salt concentration of 500 mM. After the incubation period, the extension of both the colony and the clear area around it were measured to calculate the solubilization index, as indicated above. The test was conducted in triplicate and plates with the specific substrate but without NaCl were used as the negative control.

### 2.5. Statistical Analysis

The results of all the experiments were analyzed with the Statistix v. 8.10 package (Analytical Software, USA). The effect of the endophytic strain on the enzymatic production was evaluated through a one-way ANOVA. The effect of the salt concentration on mycelial growth and the evaluation of the enzyme production under salt conditions were analyzed through a mixed-design analysis of variance (split plot ANOVA). Furthermore, this was achieved by considering the NaCl content and the fungal strain as the main and subplot factors, respectively. A Fisher’s protected least significant difference (LSD) test for multiple comparison was used when significant differences (*p* < 0.05) were found in the tests.

## 3. Results

### 3.1. Evaluation of Extracellular Enzymatic Activity

The selection of endophytes included strains of some of the most representative genera of fungi, such as *Acremonium, Didymella, Drechslera, Fusarium, Penicillium* or *Podospora*, among others. The screening of their extracellular enzymatic activity showed that all the isolates produced at least two of the enzymes (Table 2). The endophytes that produced a wider range of enzymes were E064 (*Drechslera biseptata*), E198 (*Microdiplodia hawaiiensis*), and E635 (*Penicillium chrysogenum*), which produced four types of enzymes, three of them being in common: amylases, cellulases, and lipases.

Regarding the observed frequency for each enzyme, lipase activity was the most frequent, being found in nine of the eleven strains (81.82%); only in E168 (*Fusarium avenaceum*) and E224 (*Colletotrichum cereale*) were these not detected. The endophyte E586 showed the highest solubilization index (SI = 2.07), followed by E178 (*Acremonium implicatum*) with SI = 1.90. As the second most common enzymatic activity, cellulase activity was detected in 8 out of the 11 isolates. In this case, the endophytes E586 (Unidentified sp1) and E198 (*Microdiplodia hawaiiensis*) presented the highest values (Table 2). Protease and amylase activity was found in 4 out of the 11 isolates in both cases. The endophytes E178 (*Acremonium implicatum*) and E635 (*Penicillium chrysogenum*) produced the highest protease and amylase activity, respectively. Finally, only three endophytes of the selected fungi produced either laccase (E138, *Embellisia leptinellae*; E528, *Didymella exitialis*; and E635, *Penicillium chrysogenum*) or pectinase (E138, *Embellisia leptinellae*; E198, *Microdiplodia hawaiiensis*; and E532, *Didymella phacae*) activity.

### 3.2. Halotolerance Test

**Figure 2:**
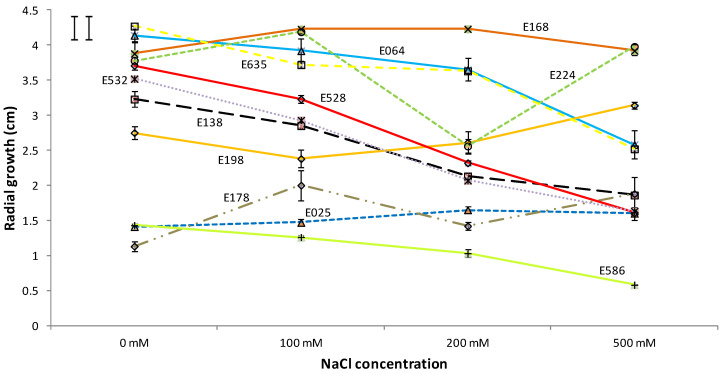
Influence of the NaCl concentration on the radial growth of the studied endophytes after 9 days of incubation. The results are given as mean ± standard error (n = 3). The least significant difference (LSD) of the salt concentration*isolate interaction is shown in the upper left corner (critical value for comparison of the same and different levels of salt are shown as left and right bars, respectively).

The growth response to saline treatments varied widely depending on the fungal species, as can be observed in Figure 2. Thus, after nine days, two of the eleven strains, E178 (*Acremonium implicatum*) and E198 (*Microdiplodia hawaiiensis*), showed a significantly higher radial growth in 500 mM of NaCl than those which were found when grown without salt stress. At the same time, two of the fungi, E168 (*Fusarium avenaceum*) and E224 (*Colletotrichum cereale*), showed higher growth under saline conditions, but only at the lower values of NaCl concentrations. On the other hand, six of the isolates (E064, E138, E528, E532, E586, and E635) showed reduced growth when they were incubated in the medium with the highest concentrations of NaCl. In any case, the eleven strains were able to grow, even under harsh saline conditions.

### 3.3. Evaluation of Enzymatic Activity under Salt Stress Conditions

This experiment showed that the solubilization index significantly changed with the salinity of the culture medium for all the parameters, except with respect to the amylolytic activity (Table 3). The production of amylase was not significantly affected by the salt concentration in any of the isolates (as the salt concentration*endophyte interaction did not significantly affect the production either), with values that ranged between 1.65, for the strain E635 (*Penicillium chrysogenum*) growing in the non-saline medium, and 2.72, for the strain E064 (*Drechslera biseptata*).

Regarding the other enzymes, the effect of salt concentration on the solubilization index changed significantly depending on the fungal strain. In the case of cellulase, four of the eight bioactive strains that produced it (E198, E224, E586 and E635) did not reduce such activity when growing in a saline medium. A better trend was found for the lipolytic activity, where 89% of the strains, i.e., all of them other than *Didymella phacae* (E532), maintained the production of this enzyme under salt conditions. In the case of pectinase production, the result for the fungus E198 (*Microdiplodia hawaiiensis*) stood out, since it was able to significantly increase its activity under saline growth conditions (with solubilization indexes of 1.33 and 1.83 for the control and salt-amended media, respectively). The same result was found in the case of proteolytic activity for the endophytes E025 (*Sarocladium terricola*) and E064 (*Drechslera biseptata*), with significant increases of 23% and 15%, respectively, when compared with the corresponding controls without salt added. Only in regard to laccases did the salt conditions significantly reduce the enzymatic activity for the three strains. However, even in this case, the three endophytes maintained their capacity to produce such compounds.

## 4. Discussion

All the studied fungi produced at least two of the six different enzymes that were analyzed. From them, ≈27% of the strains (3 out of 11) produced three of them, and the same percentage produced four of these compounds. The fact that none of the isolates showed the potential to produce all the tested enzymes is supported by the literature, where it is reported that this outcome is very rare [7,26]. Regarding the frequency of enzyme occurrence, lipases and cellulases were the most frequent enzymes since they were found in ≈82% and ≈72%, respectively, of the selected strains.

The high frequency of lipolytic activity may be considered an expected outcome, as endophytic fungi usually produce these compounds in order to overcome the defense mechanisms of their hosts [41]. This group of enzymes, together with proteases, facilitates the hyphal penetration of endophytes through the plant cell wall [42]. In this sense, three strains could produce both lipases and proteases, i.e., *Sarocladium terricola* (E025), *Drechslera biseptata* (E064), and *Acremonium implicatum* (E178). In addition, ten out of eleven could produce at least one of both groups of compounds. The solubilization index for lipases ranged from 1.10 for E138 (*Embellisia leptinellae*) to 2.07 for the unidentified strain E586, which are values that are quite similar to those found by other authors who worked with other fungal species [39] and who produced these kinds of enzymes. Therefore, the results obtained in the present study are promising enough to justify being further tested in other different conditions in order to maximize such a type of enzyme production.

Cellulases were produced by 80% of the isolates studied. It is known that cellulolytic activity is widespread among pathogenic and saprophytic microorganisms [43]. Endophytes can behave as both pathogens or as saprophytes at times during their biological cycle. This fact could explain the high proportion of endophytes which produced cellulases in the present study, although the type of relationship established between our fungi and plant host should be further investigated in order to confirm this. This is supported by the results of other studies, where a similar proportion of fungal endophytes producing cellulases was found [27]. Nevertheless, further studies should be performed to confirm this fact as other quite different results have also been found when analyzing other hosts, such as those recorded by Uzma et al. [44], where only ≈28% of the endophytes that were studied were capable of producing cellulase.

Amylolytic activity was found in ≈36% of the isolates, which is a lower frequency with respect to other articles in which the percentage of occurrence of this enzyme ranged from 78% to 100% [8,37,38,45]. This outcome may be related to the fact that fungal amylases occur more commonly in saprophytic genera, such as those found in *Aspergillus* and *Rhizopus* [46], which were not present in our study. Among our selection, *Penicillium chrysogenum* (E635), belonging to a genus of known saprotrophs, was the endophyte which presented a significantly higher amylase concentration, as was also observed by Fouda et al. [38]. This isolate (E635) was one of the most prolific enzyme producers in the present paper, producing four different enzymes. However, no pectinolytic ability was observed, which is in contrast to that which was observed in the aforementioned article. The proportion of isolates with pectinolytic activity in our study (≈27%) was in agreement with the results obtained by Shubba and Srinivas [27], albeit a little bit higher than the 19% that was observed in the endophytes isolated from different medicinal plants of India [44]; moreover, it was also lower than the 49% detected in the fungi that were isolated from Thai orchids [7].

Regarding laccases, only three of our isolates (≈27% of the total) produced them. This is in agreement with the common trend found in other similar studies, where the frequency of fungal endophytes producing this enzyme was also very low [27]. As pointed out by Uzma et al. [44], laccases are able to degrade lignin, which might have a detrimental effect on the plant host in terms of limiting their inter-relationships. Such a feature might be more common in saprophytic species. Therefore, the occurrence frequency of the production of laccases in endophytic species may be very low, appearing only in species that can also act as saprophytes at a specific moment of their life cycle. Therefore, the identification of three different isolates, E138 (*Embellisia leptinellae*), E528 (*Dydimella exitialis*), and E635 (*Penicillium chrysogenum*) with the potential to produce this scarce enzyme may also allow further development of its production for industrial application. In the same way, the results obtained by fungi E064 (*Drechslera biseptata*) and E198 (*Microdiplodia hawaiiensis*) should be highlighted, along with E635, due to their great versatility, as these three endophytes were able to produce four of the enzymatic groups that have been mentioned.

Regarding the halotolerance tests, the preliminary hypothesis that plants from the dehesa would be suitable for the identification of endophytes that are capable of growing in relatively harsh environments has been supported by the evidence. In this regard, ≈45% (five out of eleven) of the isolates showed greater mycelial growth in plates that were supplemented with 500 mM NaCl than in those which were in a non-saline PDA medium after nine days. In addition, and more importantly, none of the isolates ceased their growth under saline conditions. These data may also explain the higher enzymatic activity of several endophytes under high salinity conditions. This was the case of E198 (*Microdiplodia hawaiiensis*) with respect to pectinolytic activity, as well as in E025 (*Sarocladium terricola*) and E064 (*Drechslera biseptata*) for protease production. These endophytes may be the most suitable candidates in terms of producing the involved enzymes via by-products generated by other industries with saline properties, such as grape by-products, which are well-known for their salinity and sodicity problems [47], or olive oil mill waste, which may contain up to 2% of salt [48]. This enzymatic activity under saline conditions has also been observed in other endophytes, such as *Microsphaeropsis arundinis* that are isolated from mangrove trees and which showed a higher ligninolytic activity under saline conditions [49]. For the massive production of such enzymes with these endophytes, the following step may be the evaluation of their production by directly using different by-products as the growing substrate medium in order to confirm their suitability and their optimization of the growth conditions. The specific hydrolytic compounds produced and their quantification may also require further investigation. Other enzymatic activities, such as the production of amylases, although not increased, were not affected by the salinity of the media. Additionally, even in the cases where the production of enzymes was significantly reduced, such as the production of laccases in the three tested isolates, only one endophyte, *Fusarium avenaceum* (E168), lost its enzymatic activity under saline conditions. Therefore, these endophytes, although not so promising, could also be considered in further steps.

## 5. Conclusions

The experimental results evidenced that the fungal endophytes that were isolated from plants naturally present in Spanish dehesas can produce a wide range of enzymes that are greatly valued for numerous industries. Among them, some of the strains—especially *Sarocladium terricola* (E025), *Acremonium implicatum* (E178), *Microdiplodia hawaiiensis* (E198), and an unidentified species (E586)—could be suitable for the production of such enzymes under saline conditions, which may allow the utilization of by-products as growth substrates for large-scale production. However, since the enzymatic activity is somehow modulated by the substrate, this study should only be considered an initial approach, which was conducted in order to continue working on the identification of the compounds and to develop the optimization of their production by directly using those residues.

## Figures and Tables

**Table 1:** Fungal endophytes used in the experiments. For every strain, the internal code used in the laboratory, the plant host where it was isolated from, the tentative identification, and the Genbank accession number are shown.

Code	Plant Host	Identification ^1^	Genbank Accession #
E025	*Biserrula pelecinus*	*Sarocladium terricola (J.H. Mill., Giddens & A.A. Foster)*	OK161081
E064	*Poa annua*	*Drechslera biseptata* (Sacc. & Roum.) M.J. Richardson & E.M. Fraser	KP698352
E138	*Ornithopus compressus*	*Embellisia leptinellae* E.G. Simmons & C.F. Hill	KP698337
E168	*Trifolium subterraneum*	*Fusarium avenaceum* (Fr.) Sacc.	KP698339
E178	*Poa annua*	*Acremonium implicatum* (J.C. Gilman & E.V. Abbott) W. Gams	KP899410
E198	*Ornithopus compressus*	*Microdiplodia hawaiiensis* Crous	KP899392
E224	*Lolium rigidum*	*Colletotrichum cereale* Manns	KP698357
E528	*Lolium rigidum*	*Didymella exitialis* (Morini) E. Müll.	KP899390
E532	*Medicago polymorpha*	*Didymella phacae* Corbaz	KP698363
E586	*Bromus mollis*	Unidentified sp1	KP899447
E635	*Medicago polymorpha*	*Penicillium chrysogenum* Thom	KP899406

^1^ Based on morphological characteristics and by comparison with ITS sequences in GenBank and UNITE (version 8.3), with a similarity ≥99%.

**Table 2:** Solubilization index (mean ± standard error) produced by each endophyte for each enzyme. A summary of the ANOVA, showing the effect of the endophyte, with the DF (degree of freedom) and F values, including the level of significance (*** *p* ≤ 0.001), indicated in the second row for each enzyme activity.

Endophyte	DF	Amylase	Cellulase	Laccase	Lipase	Pectinase	Protease
	10	51.64 ***	52.84 ***	781.87 ***	37.69 ***	7362.28 ***	541.19 ***
E025		WA	WA	WA	1.55 ± 0.13 c	WA	1.12 ± 0.02
E064		1.27 ± 0.04 b	1.38 ± 0.14 bc	WA	1.56 ± 0.25 c	WA	1.28 ± 0.08
E138		WA	WA	1.16 ± 0.02 b	1.10 ± 0.01 d	1.17 ± 0.00 a	WA
E168		WA	1.39 ± 0.05 bc	WA	WA	WA	1.05 ± 0.00 c
E178		WA	WA	WA	1.90 ± 0.09 ab	WA	1.49 ± 0.03 a
E198		1.34 ± 0.06 b	2.27 ± 0.20 a	WA	1.23 ± 0.03 d	1.17 ± 0.02 a	WA
E224		1.30 ± 0.05 b	2.05 ± 0.09 a	WA	WA	WA	WA
E528		WA	1.63 ± 0.09 b	1.36 ± 0.06 a	1.67 ± 0.19 bc	WA	WA
E532		WA	1.22 ± 0.06 c	WA	1.19 ± 0.05 d	1.10 ± 0.01 b	WA
E586		WA	2.31 ± 0.28 a	WA	2.07 ± 0.07 a	WA	WA
E635		1.78 ± 0.33 a	1.33 ± 0.01 bc	1.09 ± 0.02 c	1.19 ± 0.02 d	WA	WA

WA: without activity. In the cases with positive activity, means in the same column with different letters are significantly different according to the LSD test. Three repetitions were performed for each treatment (n = 3).

**Table 3:** Enzymatic production under different salt conditions by the selected strains. The positive results (mean ± se) are shown as the solubilization index ((colony + halo zone diameters)/colony diameter). A summary of the ANOVA (DF: degree of freedom; F values; and level of significance (** *p* ≤ 0.01, *** *p* ≤ 0.001)), showing the effect of the endophyte, the salt concentration, and their interaction, is shown for each parameter.

Source		Amylase	Cellulase	Laccase	Lipase	Pectinase	Protease
Salt (S)	DF	1	1	1	1	1	1
	F	0.02	231.60 ***	296.22 ***	13.90 **	24.75 ***	62.71 ***
Endophyte (E)	DF	3	7	2	8	2	3
	F	33.52 ***	435.09 ***	108.34 ***	195.6 ***	56.85 ***	151.67 ***
S*E	DF	3	7	2	8	2	3
	F	2.23	43.57 ***	27.90 ***	7.37 ***	111.59 ***	198.63 ***
E	S	Amylase	Cellulase	Laccase	Lipase	Pectinase	Protease
E025	0 mM	WA	WA	WA	2.58 ± 0.14 b	WA	1.78 ± 0.03 d
	500 mM	WA	WA	WA	2.53 ± 0.15 b	WA	2.19 ± 0.06 bc
E064	0 mM	2.72 ± 0.20	1.33 ± 0.04 ef	WA	3.29 ± 0.18 a	WA	2.22 ± 0.05 b
	500 mM	2.46 ± 0.03	1.09 ± 0.02 h	WA	3.08 ± 0.25 a	WA	2.56 ± 0.10 a
E138	0 mM	WA	WA	2.09 ± 0.01 a	1.12 ± 0.02 g	2.08 ± 0.02 b	WA
	500 mM	WA	WA	1.52 ± 0.03 b	1.07 ± 0.01 g	2.04 ± 0.02 b	WA
E168	0 mM	WA	1.41 ± 0.03 e	WA	WA	WA	2.22 ± 0.09 b
	500 mM	WA	0.00 ± 0.00 j	WA	WA	WA	0.00 ± 0.00 e
E178	0 mM	WA	WA	WA	2.25 ± 0.05 c	WA	2.01 ± 0.09 c
	500 mM	WA	WA	WA	2.03 ± 0.03 c	WA	1.62 ± 0.02 d
E198	0 mM	2.71 ± 0.10	2.61 ± 0.03 b	WA	3.27 ± 0.10 a	1.33 ± 0.05 e	WA
	500 mM	2.65 ± 0.10	2.58 ± 0.08 b	WA	3.23 ± 0.08 a	1.83 ± 0.06 c	WA
E224	0 mM	2.44 ± 0.06	2.59 ± 0.07 b	WA	WA	WA	WA
	500 mM	2.48 ± 0.09	2.52 ± 0.06 b	WA	WA	WA	WA
E528	0 mM	WA	2.86 ± 0.12 a	1.48 ± 0.03 b	1.48 ± 0.03 def	WA	WA
	500 mM	WA	2.28 ± 0.09 c	1.30 ± 0.02 c	1.30 ± 0.02 efg	WA	WA
E532	0 mM	WA	1.25 ± 0.03 fg	WA	1.23 ± 0.03 fg	2.35 ± 0.07 a	WA
	500 mM	WA	0.46 ± 0.04 i	WA	0.17 ± 0.01 h	1.48 ± 0.04 d	WA
E586	0 mM	WA	1.88 ± 0.06 d	WA	1.72 ± 0.03 d	WA	WA
	500 mM	WA	2.00 ± 0.02 d	WA	1.48 ± 0.03 def	WA	WA
E635	0 mM	1.65 ± 0.04	1.2 ± 0.03 fgh	2.13 ± 0.06 a	1.33 ± 0.03 efg	WA	WA
	500 mM	1.90 ± 0.02	1.12 ± 0.03 gh	1.50 ± 0.02 b	1.55 ± 0.02 de	WA	WA

WA: without activity. In the cases with positive activity, means in the same column with different letters are significantly different according to the LSD test. Three replicates were performed for each treatment (n = 3).

## Data Availability

All data are included in the present study.
